# A Confirmatory Factor Analysis of the ‘Return to Duty Readiness Questionnaire’

**DOI:** 10.3390/healthcare11010041

**Published:** 2022-12-23

**Authors:** Carly Cooper, Bruce Frey, Haiying Long, Charles Day

**Affiliations:** 1 Department of Occupational Therapy, Brooke Army Medical Center, Fort Sam Houston, San Antonio, TX 78234, USA; 2 Department of Educational Psychology, University of Kansas, Lawrence, KS 66045, USA; 3 Irwin Army Community Hospital, Soldier Centered Medical Homes, Fort Riley, KS 66442, USA

**Keywords:** Return to Duty Readiness Questionnaire, military, factor structure, musculoskeletal pain

## Abstract

The Readiness to Return to Duty Questionnaire (RDRQ) is a recently developed screening instrument for detecting fear-avoidance behavior in a military musculoskeletal pain population. The RDRQ was developed based on the Fear-Avoidance Model which postulates four factors resulting in overall fear-avoidance behavior. While research investigating the factor structure of the RDRQ does not exist, research investigating the factor structure of other measures of fear avoidance have found evidence of one and two factor solutions. In the present paper we assess the adequacy of the proposed factor structure of the RDRQ using confirmatory factor analysis. The results favor a three-factor model. Theoretical implications for research using the RDRQ are discussed.

## 1. Introduction

Pain is a complex phenomenon that may not be effectively treated using a unidimensional approach [1]. The U.S. Army Holistic Health and Fitness initiative postures that holistic health is a multidimensional concept, and as a result, has adapted a health initiative that makes educative, nonpharmacological, and holistic approaches to pain management a priority in preventing pain- related disability [1,2]. A result of these holistic movements is the development of the Return to Duty Readiness Questionnaire (RDRQ), based on the Fear Avoidance Model (FAM), as a fear avoidance screening for active-duty service members receiving care for musculoskeletal pain. The FAM has gained popularity since its initial inception as a multidimensional model to explain the development of chronic pain and association with pain-related disability [3]. The relationship between fear and pain introduced by Lethem et al. (1983) proposed that individuals sustaining a painful injury will either confront the pain or avoid the pain [4]. Four main components are at the core of Lethem’s concept: Catastrophizing, Fear of Pain, Kinesophobia (fear of movement or re-injury), and Avoidance Behavior. Vlaeyen and colleagues took Lethem’s concept to develop and introduce a cognitive behavioral Fear Avoidance Model [5].

The Return to Duty Readiness Questionnaire scale was developed in 2019 to fill the measurement gap of fear avoidance behavior assessment in the military [6]. The item development process involved creating items from the above scales, using a systematic with the four dimensions (avoidance behavior, fear of pain, kinesiophobia and catastrophizing), using a panel of subject matter military medicine experts. Appendix A outlines the RDRQ items and the specifications of each item in relation to each construct theoretical framework. Initial validation of the RDRQ found the instrument to be valid through establishing convergent validity with the Fear Avoidance Components Scale [7] (rho = 0.74, *p* < 0.001) and reliable via internal consistency estimation (α = 0.94, Ω = 0.96) with a population of active-duty service members [6]. Further psychometric evidence of the RDRQ through evaluation of factor structure will guide further development and theory supporting fear-avoidance assessment in the military. While research investigating the factor structure of the RDRQ does not exist, research investigating the factor structure of other measures of fear avoidance have found evidence of one and two factor solutions. Prior research exploring the factor structure of the FACS, which was also developed based on the four constructs of the Fear Avoidance Model, has yielded results that are consistent with a two-factor structure instead of the four for those samples including the English version and several language adapted versions [7,8,9,10].

For factor structures that have already been defined, confirmatory factor analysis (CFA) is a method to verify such factors through use of model fit analysis [11]. In contrast to exploratory factor analysis, this specified model specifies which variables load onto certain factors, as well as which factors are correlated [12]. Since the RDRQ was developed with a strong theoretical foundation, the confirmatory factor analysis methodology was used to provide a robust assessment of the factor structure of the measure. This study sought to answer the question of whether the internal structure analysis of the RDRQ supports the instrument’s Fear Avoidance Model four factor theory. 

## 2. Materials and Methods

### 2.1. Sample and Procedure

A convenience sample of 240 active-duty service members from one Soldier Centered Medical Clinic completed questionnaires over a three-year period. The questionnaire is collected as part of the standard of care clinical intake packet. Only questionnaires collected from January 2020 to May 2022 were given to the researcher for analysis. The participating clinic was chosen because its active-duty customer base is representative of the intended instrument population. Participants were required to complete the RDRQ as part of the normal operational intake packet completed upon initial appointment with the clinic provider. Completed questionnaires were delivered directly to the researchers upon request. There were no missing RDRQ responses as the collection site only delivered questionnaires that were complete. Demographics show that 90% of respondents are of enlisted military rank. Mean age of respondents was 29.33 years (s = 7.43, min = 19, max = 49). The age range most represented was that of the 21–25 years, and as expected, the male gender is most represented (28% female, 72% male). The Military Occupational Specialty (MOS) most represented (*n* = 45) in the data was 31B Military Police. 

Institutional IRB approval was granted before any research activity was initiated. Demographic information collected in a way to protect participant Protected Health and Individual Information as to not be able to identify participant based on responses. Informed consent was not collected as these questionnaires were a part of standard of care at this clinic and protected information was not being collected. 

### 2.2. Measures

The RDRQ (Appendix A) was designed to measure the level of fear-avoidance in military (specifically U.S. Army) samples. The 20 item self-report consists of response options that range from 1 (‘Not at All’) to 5 (‘Completely Agree’). Respondents are asked to respond to questions aimed at evaluating the level of fear-avoidance behavior based on questions derived from four categories theorized to compose the fear-avoidance construct. High scores indicate higher level of fear-avoidance behavior. Upon initial validation, the RDRQ was found to have an acceptable composite level internal consistency reliability (Cronbach’s alpha coefficient and McDonald’s omega coefficient): 0.94 and 0.96, respectively [6]. 

### 2.3. Statistical Analyses

**Figure 1:**
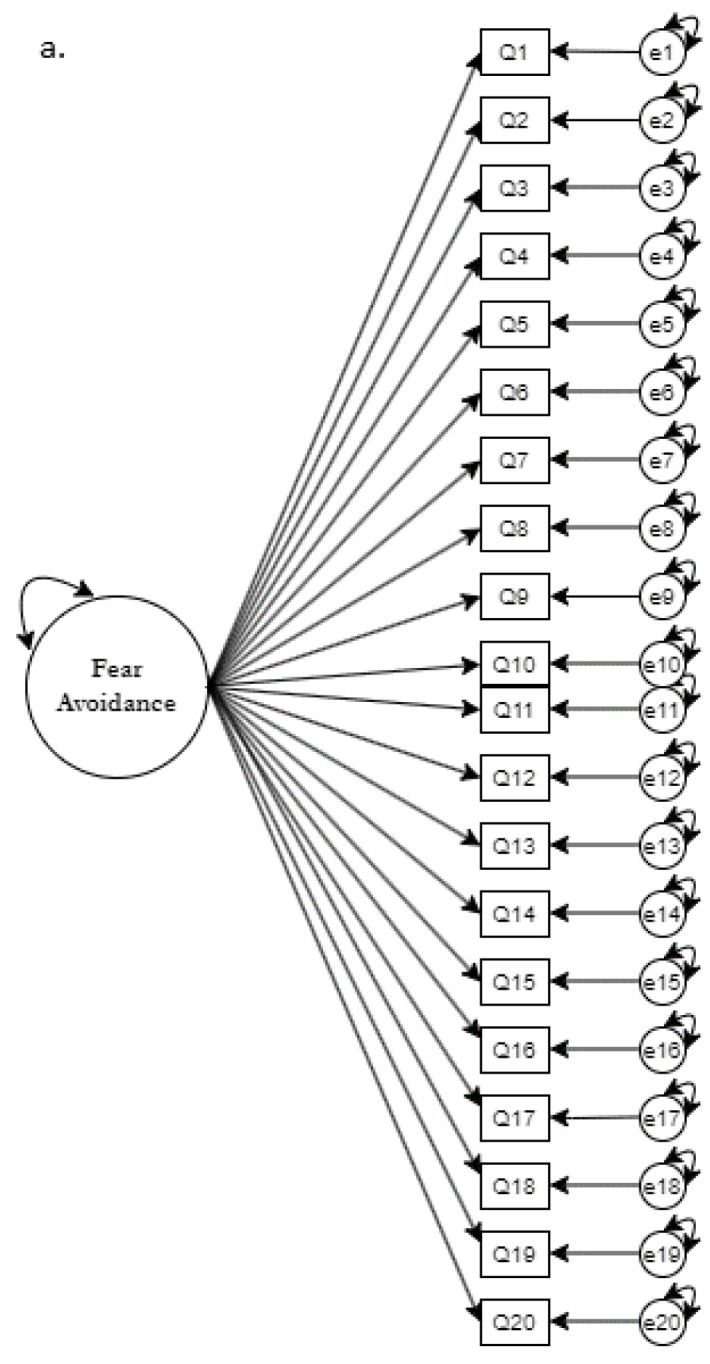

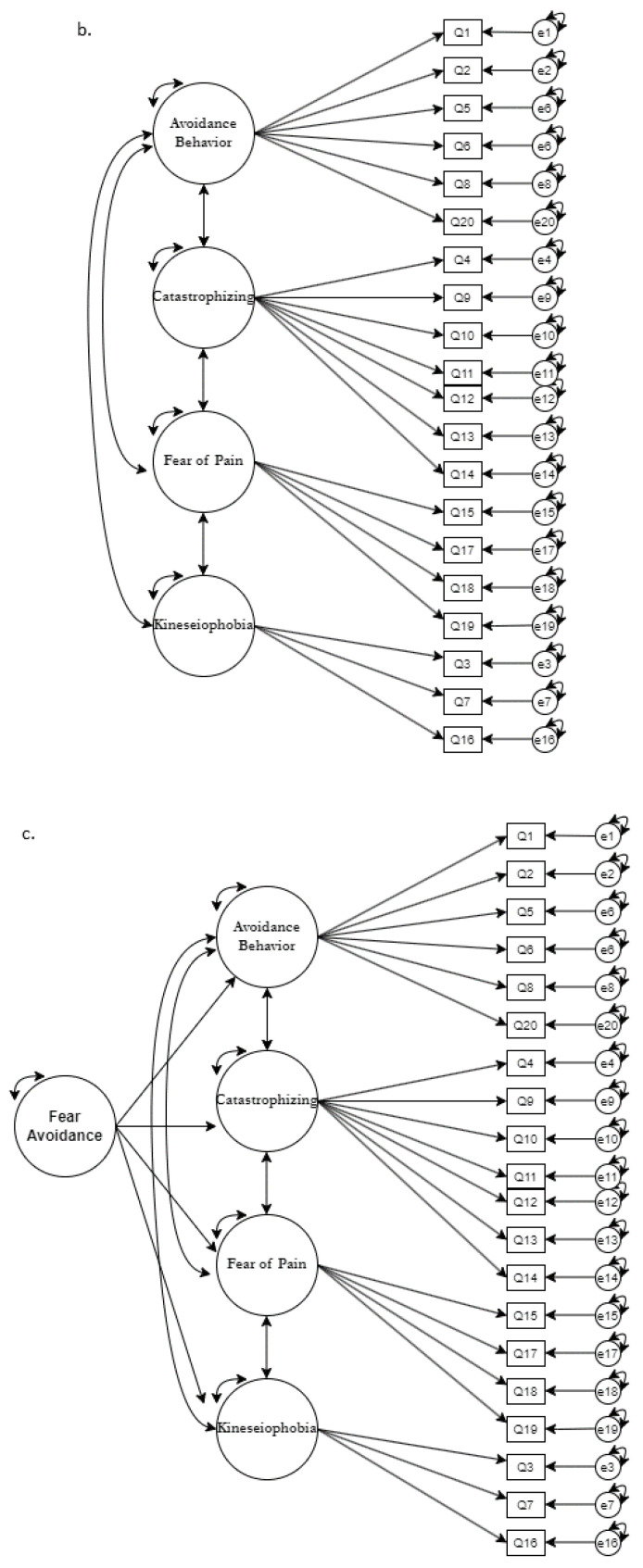
Competing Confirmatory Factor Analysis Models: (**a**) base model, (**b**) four-factor model, (**c**) second-order model.

The factor structure of the RDRQ was tested through confirmatory factor analysis using R 4.1.1 with the following packages: ‘readxl’, ‘foreign’, and ‘lavaan’ [13,14,15]. Structural equation modeling techniques allow specification of the number of dimensions (factors) in a model, as well as the items expected to load on each dimension. The hypothesized relationships are tested empirically for goodness of fit with the sample data. Model global fit was evaluated based on the following common fit indices: chi-square absolute fit (*p* > 0.05), standardized root means square (SRMR < 0.08), root means square error of approximation (RMSEA < 0.08), comparative fit index (CFI > 0.95), and Tucker–Lewis index (TLI > 0.90) [12]. Local fit was determined through item analysis and factor correlations [11]. Figure 1 shows the comparisons of competing A base model was identified and evaluated by loading all indicators onto one factor, presumed to be the overall fear avoidance latent variable. Model fit was further assessed via first order CFA to test the four-factor theory driven item dimensions that compose the FAM. Second order CFA structure was analyzed due to the correlation between the four first order factors being caused by the overarching fear avoidance factor [16]. Any model modifications deemed necessary through factor loadings and model fit analysis were carefully constructed through analysis suggestion and theoretical basis [12]. All analyses conducted used maximum likelihood estimation with robust standard errors (MLR). A base model was identified and evaluated by loading all indicators onto one factor, presumed to be the overall fear avoidance latent variable. Model fit was further assessed via first order CFA to test the four-factor theory driven item dimensions that compose the FAM. Second order CFA structure was analyzed due to the correlation between the four first order factors being caused by the overarching fear avoidance factor [16]. Model modifications were carefully constructed through analysis software suggestion and theoretical basis. 

## 3. Results

A total of 240 RDRQ surveys that were collected were given to the researcher for analysis. There were no missing RDRQ responses as the collection site only delivered questionnaires that were complete. Mean values for the items ranged from 1.09 to 2.52, with a mean RDRQ total score of 38.53 (SD = 17.51). A high standard deviation suggests a wide variability of fear-avoidance in this sample. Table 1 shows the mean, skewness and kurtosis for each RDRQ item indicating acceptability for utilizing structured equation modeling. 

Figure 1 shows items included in the RDRQ according to which factor they are being specified to belong. Questions were assigned to factors through item development from the panel of Subject Matter experts and by adaptation of items from other fear-avoidance questionnaires also based on the Fear-Avoidance Model [7]. Before testing the theoretically driven four factor model, a unidimensional factor model was tested to compare whether the proposed structure was in fact best represented by multiple factors instead of just one. After those two models were tested, a higher order model was tested to determine if this data may perhaps be best represented with an overarching general factor influencing the four theoretical models. Using model fit criteria, the best fitting model was chosen for further respecification and refinement. 

**Figure 2:**
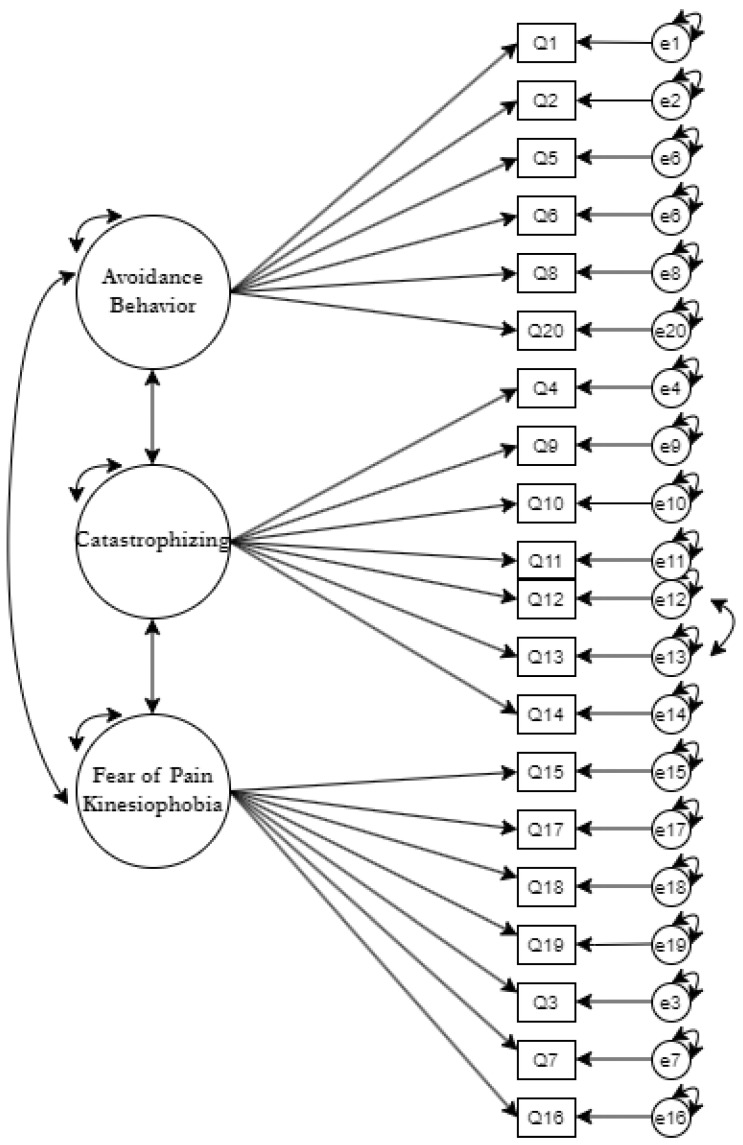
Best Fit Confirmatory Factor Analysis Model: Three-factor model.

In examining hypothesized structures of military fear avoidance, a series of three latent variable models was fit: a unidimensional model, a four-factor model, and a higher order model. Goodness of fit statistics was not the only consideration. Theoretical construct, model parsimony, and chi-square difference tests were also considered. Thus, it was determined that the four-factor model was the best candidate for model respecification and refinement. To begin model respecification for the four-factor model, a warning message referencing too high of a correlation between FOP and KIN needed to be addressed. A series of three modified versions of the model with three factors: AB, CAT, FOP_KIN was executed. The third model generated the most acceptable global fit indices while remaining parsimonious as outlined in Table 2. This final model includes the following modifications to the original four factor model: FOP and KIN combined, RDRQ3 moved to CAT factor; correlated errors specified for RDRQ12 with RDRQ13. This model is visualized in Figure 2. 

## 4. Discussion

This study assessed for the first time the factor structure of the RDRQ in active-duty U.S. Army service members. Confirmatory factor analysis, for this sample data, did not support any of the originally hypothesized conceptual foundations of the RDRQ. Of the three originally proposed models, (one factor, four factor, higher order) the four-factor model was chosen for further respecification and refinement. This decision was made based on three outcomes. The fear avoidance model theory that the instrument was founded on, the parsimony of the model compared to a higher order model, and the overall global fit with chi-square difference testing calculations. After further model refinement, the best fitting and most parsimonious result revealed a model in which the FOP and KIN factors were combined, item RDRQ3 was reassigned; although still demonstrating CFI and TLI estimations not meeting identified fit values. This is shown in Figure 2 with item pairs RDRQ12/RDRQ13 allowed correlated errors. The results of the best fitting four factor model CFA still resulted in many items below the 0.50 level indicating that the items are not responsible for explaining a majority of the variance associated with their factor and should be investigated further [12]. 

Examination of the items that made up the FOP and KIN factors that were ultimately combined, suggest a new factor that evokes pain-related anxiety related to return to work. Perhaps, with the combined factor, results in three factors (1) Avoidance Behavior, (2) Catastrophizing, and (3) Work Anxiety. The two combined factors, FOP and KIN, are defined similarly so those items will likely be written and interpreted similarly. Fear of pain (FOP) is commonly defined as an abnormal or persistent fear of physical pain, while kinesiophobia (KIN) is pain-related fear of movement [17,18]. In either case, pain and anxiety associated with both is acting as a protective measure. 

### 4.1. Limitations

While the sample size meets requirements for minimum to conduct confirmatory factor analysis, a larger sample size with a broader range of MOS would yield more generalizability evidence. The collection site also did not discern between initial visits vs. follow up visits, or acute vs. chronic pain for who was given the scales for completion. The RDRQ items were adapted from existing fear-avoidance questionnaires that have been studied mainly on chronic pain populations. The RDRQ may be completed differently by those being seen for an acute condition versus chronic or becoming chronic condition that was not designated at the collection site. Validation of the items in question, especially the items regarding leadership would benefit from deliberate questionnaire administration via therapist/researcher/primary provider. Providing information related to the impact of completing the questionnaire on respondent health care decisions may impact the thought and time going into questionnaire response. This could ultimately affect future study of response process validity.

### 4.2. Future Directions & Recommendations

The results of the confirmatory factor analysis offer an interesting avenue to contribute to the Fear Avoidance Model theory and how it relates to the military population. RDRQ developers are encouraged to explore the combined FOP and KIN factors, possibly creating, and renaming a new factor specific to the military as a modification to the Fear Avoidance Model for this population. Kline recommends seriously considering the removal of indicators that have less than 50% of the variance explained due to the factor [12]. This would include consideration of removing those items specifically referenced at the beginning of the discussion section after evaluating the necessity of each to re-examine the original models and the successful three factor model found here. Additional investigation of the three-factor model that was found to be the best and most parsimonious model using the data for is recommended. This model should be investigated on different military populations such as training units and non-infantry posts to add to the validation and justification of the measurement tool. Larger sample sizes are required to conduct any measurement invariance testing. This may offer additional insight into items that are more susceptible to certain rank groups or gender, providing further validity evidence based on internal structure. Another future analyzing method is the use of parceling; an average across a set of homogeneous items such as with a Likert scale [12].

## 5. Conclusions

This study suggests that the Return to Duty Readiness Questionnaire (RDRQ) measures factors of fear avoidance and can be used to assess fear avoidance behavior in a military musculoskeletal pain population although additional scale revision and study replication is needed to make a definitive conclusion regarding the structure of the RDRQ. Evidence for a unidimensional latent variable or higher order model was not supported with these data. The structure of the RDRQ reflects three separate but related factors of avoidance behavior. Although global model fit for this structure was acceptable, the model fails to explain the majority variance for 8 of the 20 indicators. Future internal factor evaluation using a three-factor solution should be studied in additional populations. 

## Figures and Tables

**Table 1:** RDRQ Item Means, Skewness, and Kurtosis.

Items	N	M	sd	Skewness	Kurtosis
RDRQ1	240	1.91	1.24	1.16	0.14
RDRQ2	240	2.50	1.49	0.48	−1.20
RDRQ3	240	1.92	1.29	1.25	0.31
RDRQ4	240	2.26	1.23	0.56	−0.14
RDRQ5	240	1.71	1.14	1.57	1.45
RDRQ6	240	2.02	1.38	1.12	−0.11
RDRQ7	240	2.01	1.33	1.11	−0.06
RDRQ8	240	2.00	1.21	1.06	0.18
RDRQ9	240	2.28	1.35	0.75	−0.66
RDRQ10	240	1.85	1.21	1.33	0.73
RDRQ11	240	1.40	0.84	2.53	6.43
RDRQ12	240	1.58	1.09	1.87	2.46
RDRQ13	240	1.68	1.18	1.70	1.79
RDRQ14	240	1.44	1.01	2.38	4.70
RDRQ15	240	2.34	1.41	0.67	−0.89
RDRQ16	240	2.42	1.37	0.67	−0.72
RDRQ17	240	2.12	1.30	1.05	−0.05
RDRQ18	240	1.78	1.11	1.33	0.80
RDRQ19	240	2.32	1.36	0.77	−0.62
RDRQ20	240	2.03	1.33	1.13	0.04

**Table 2:** Global Fit Indices for CFA Models.

Model	χ^2^	df	CFI	TLI	RMSEA	SRMR	χ^2^ Difference
Unidimensional	504.931	170 *p* = 0.000	0.834	0.815	0.091 *p* = 0.000 90% CI [0.083—0.098]	0.067	0.000028 *p* < 0.001
Four Factor Model	427.121	164 *p* = 0.000	0.870	0.849	0.082 *p* = 0.000 90% CI [0.074—0.090]	0.066	
Second Order Model	439.631	169 *p* = 0.000	0.855	0.849	0.082 *p* = 0.000 90% CI [0.074—0.090]	0.076	0.02761 *p* = 0.050
Respecified Three-Factor Model	375.226	166 *p* = 0.000	0.897	0.882	0.072 *p* = 0.000 90% CI [0.063—0.080]	0.060	<0.00001 *p* < 0.001

Note. All significance levels set to *p* = 0.050.

**Figure A1:**
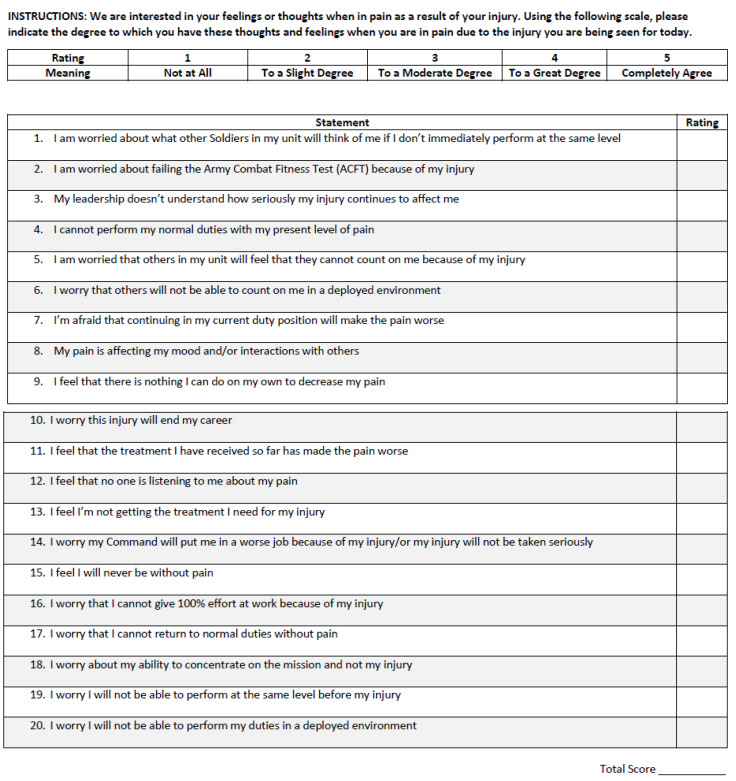
Return to Duty Readiness Questionnaire (RDRQ).

## Data Availability

Data used in this study can be found at https://www.kaggle.com/datasets/carlycooper/return-to-duty-readiness-questionnaire-responses accessed on 15 September 2022 R code used for data analysis can be found at https://www.kaggle.com/code/carlycooper/notebooke046c1872a/edit accessed on 2 December 2022.
